# Attachment and the Development of Depressive Symptoms in Adolescence: The Role of Regulating Positive and Negative Affect

**DOI:** 10.1007/s10964-021-01426-y

**Published:** 2021-04-01

**Authors:** Martine W. F. T. Verhees, Chloë Finet, Sien Vandesande, Margot Bastin, Patricia Bijttebier, Nadja Bodner, Tanya Van Aswegen, Magali Van de Walle, Guy Bosmans

**Affiliations:** 1 Clinical Psychology, KU Leuven, Leuven, Belgium; 2 Clinical Child and Family Studies, Vrije Universiteit Amsterdam, Amsterdam, The Netherlands; 3 School Psychology and Development in Context, KU Leuven, Leuven, Belgium; 4 Parenting and Special Education, KU Leuven, Leuven, Belgium; 5 Quantitative Psychology and Individual Differences, KU Leuven, Leuven, Belgium; 6 Department of Psychiatry, Stellenbosch University, Stellenbosch, South Africa; 7 Clinical Psychology, Vrije Universiteit Amsterdam, Amsterdam, The Netherlands

**Keywords:** Attachment, Depressive symptoms, Emotion regulation, Adolescence

## Abstract

Although widely accepted, attachment theory’s hypothesis that insecure attachment is associated with the development of depressive symptoms through emotion regulation strategies has never been longitudinally tested in adolescence. Additionally, previous research only focused on strategies for regulating negative affect, whereas strategies for regulating positive affect may also serve as a mechanism linking insecure attachment to depressive symptoms. This study aimed to fill these research gaps by testing whether the association between attachment and change in depressive symptoms over time is explained by strategies for regulating negative and positive affect in adolescence. Adolescents (*N* = 1706; 53% girls; *M*_age_ = 12.78 years, SD_age_ = 1.54 at Time 1) were tested three times, with a 1-year interval between measurement times. They reported on their attachment anxiety and avoidance at Time 1, depressive symptoms at Times 1 and 3, and regulation of negative affect (brooding and dampening) and positive affect (focusing and reflection) at Time 2. The results from multiple mediation analyses showed that more anxiously attached adolescents developed more depressive symptoms via increased brooding and dampening. More avoidantly attached adolescents developed more depressive symptoms via decreased focusing. These findings provide longitudinal support for attachment theory’s emotion regulation hypothesis, and show that the regulation of both negative and positive affect is important.

## Introduction

Depressive symptoms are among the most prevalent psychological problems in adolescence, with a steep increase in symptoms during the transition from childhood to adolescence (Thapar et al., 2012). Because many episodes of adult depression have their onset in adolescence, identifying risk factors that predict the development of adolescent depression is critical to understand the long-term increased vulnerability for recurrent depression (Hankin, 2015). Insecure attachment is an important factor predicting depressive symptoms (Spruit et al., 2020). It has been proposed that emotion regulation strategies underlie the association between insecure attachment and depressive symptoms, but this has never been longitudinally tested in adolescence (Malik et al., 2015). This marks a research gap since cross-sectional research does not allow examining temporal pathways and cross-sectionally tested mediation effects are at risk to provide biased estimates (e.g., Maxwell et al., 2011). What is more, existing studies solely focused on regulation of negative affect as a mechanism linking attachment to depressive symptoms and did not include strategies for regulating positive affect. However, attachment may also be related to the regulation of positive emotions, which in turn may be associated with the development of depressive symptoms (e.g., Gentzler et al., 2010; Raes et al., 2009). This study aimed to fill these existing research gaps by longitudinally testing whether the association between insecure attachment and development of depressive symptoms over time is explained by strategies for regulating negative and positive affect in adolescence.

### Attachment and Depressive Symptoms

According to attachment theory, children who repeatedly experience sensitive and responsive parental caregiving develop secure attachment representations (Bowlby, 1969). These children’s representations reflect confident expectations that they are worthy of receiving care and that caregivers will support their ability to explore their environment as well as provide support in times of distress (Cassidy, 2016). Children who repeatedly experience insensitive and unresponsive caregiving develop less secure attachment representations and lack the confidence that the caregiver will be available to provide care and support when needed. When the quality of parental support is unpredictable this can lead to increased anxiety about the availability of support (attachment anxiety); when parents are perceived as unavailable or rejecting this can lead to the expectation that it is better to avoid support-seeking (attachment avoidance; Cassidy, 2016).

Meta-analyses have shown that insecure attachment is associated with depressive symptoms in adults and in children and adolescents (Dagan et al., 2018; Spruit et al., 2020). Moreover, longitudinal research showed that insecure attachment during early adolescence predicts the development of depressive symptoms later in adolescence (Bosmans, Van de Walle et al., 2020). However, less is known about the mechanisms that explain this longitudinal association. Different attachment-related characteristics have been proposed to underlie the increased vulnerability of insecurely attached individuals for developing depressive symptoms (Brumariu & Kerns, 2010). Examples are maladaptive and biased cognitions and increased susceptibility to interpersonal stressors (Hankin et al., 2005; Morley & Moran, 2011). Important for the present study, attachment researchers increasingly point to the importance of impaired emotion regulation skills as a mechanism underlying the association between attachment and depressive symptoms (e.g., Malik et al., 2015).

### Attachment and Emotion Regulation

Emotion regulation involves the monitoring, evaluation and modulation of emotional responses (Contreras & Kerns, 2000). Emotion regulation encompasses responses to both negative and positive affect. Theory and research indicate that emotion regulation strategies develop largely within the co-regulating parent-child attachment relationship (Brumariu, 2015). Securely attached children’s repeated interactions with sensitive and responsive parents foster their ability to seek support during distress, to openly express and share their emotions and to effectively use the parent to help co-regulate their emotions when needed (Mikulincer et al., 2003; Shaver & Mikulincer, 2002). Moreover, these children receive more instruction and modeling of appropriate emotion regulation strategies (Brumariu, 2015). As a result, they develop more adaptive emotion regulation skills that allow them to actively regulate negative affect and benefit from positive affect (Mikulincer et al., 2003). In contrast, insecure attachment is linked to decreased support-seeking during distress (e.g., Dujardin et al., 2016). Insecurely attached children develop less adaptive emotion regulation skills compared to more securely attached children (Cooke et al., 2019).

Anxious and avoidant attachment styles have been proposed to be related to different styles of regulating negative and positive affect (Cassidy, 1994). Anxious attachment is associated with heightened display of negative emotions in order to elicit care from inconsistently available caregivers (Mikulincer et al., 2003). This hyperactivation strategy relates to rumination about negative emotions, i.e., repetitively focusing on distress as well as on its potential causes and consequences (Nolen-Hoeksema et al., 2008). Indeed, rumination or brooding on negative emotions has been positively associated with attachment anxiety (e.g., Van de Walle et al., 2016). Regarding positive affect, associations between attachment anxiety and emotion regulation have been less studied. Some scholars argue that more anxiously attached children also hyperactivate positive emotions, because these may also enhance closeness to the caregiver (e.g., Mikulincer & Shaver, 2007). In contrast, others argue that these children may be motivated to minimize positive emotions, in order to emphasize their negative emotions as they expect these to elicit more care from attachment figures (Cassidy, 1994). Consistent with the latter prediction, research in adults suggested that more anxiously attached individuals are more likely to dampen positive affect (e.g., Gentzler et al., 2010).

More avoidantly attached children have been observed to minimize their emotion expression in order to avoid further parental rejection (Cassidy, 1994). Avoidant attachment is related to deactivating emotion regulation strategies, such as downplaying or suppressing negative as well as positive emotions (Mikulincer & Shaver, 2007). Previous adult research indicated higher levels of dampening positive affect for more avoidantly attached individuals (Gentzler et al., 2010). Additionally, it has been proposed that attachment avoidance is related to the non-use of adaptive positive emotion regulation strategies, i.e., more avoidantly attached individuals are less able to use positive emotions as a resource (Mikulincer et al., 2003).

### Emotion Regulation and Depressive Symptoms

Maladaptive emotion regulation strategies have been proposed to increase the risk to develop depressive symptoms when experiencing negative life events or distress (e.g., Joormann & Stanton, 2016). Although emotion regulation involves the regulation of both negative and positive emotions, research on the association between emotion regulation and depressive symptoms mainly focused on the regulation of negative emotions, i.e., how individuals respond to negative affect. One often examined strategy is rumination about negative emotions (Nolen-Hoeksema et al., 2008). Rumination has two subtypes: brooding and reflection. Brooding refers to the heightening of negative affect and means that individuals passively compare their current, upsetting situation with an unachieved standard, for example, when one is sad, one thinks about why (s)he cannot handle things better. Increased brooding has been associated with more depressive symptoms concurrently and longitudinally (Treynor et al., 2003; Verstraeten et al., 2010). Reflection refers to purposefully trying to gain insight into problems to deal with or overcome depressive feelings, for example, when one is sad one goes someplace alone to think about one’s feelings. The findings on the association between reflection and depressive symptoms are mixed, with non-significant or positive associations concurrently, but at the same time reflection has been reported to be protective in the development of depressive symptoms (Treynor et al., 2003; Verstraeten et al., 2010).

Recently there has been an increasing awareness that positive emotion regulation strategies may be equally important as negative emotion regulation strategies in the prediction of depressive symptoms (e.g., Raes et al., 2009). Two positive emotion regulations strategies that have received attention in the context of depression are dampening and focusing. Dampening refers to engaging in cognitive responses that deactivate or counter positive affect (Feldman et al., 2008). For example, thinking that when something positive happens it is undeserved or will not last. Focusing refers to the ability to focus the attention on experiences of positive affect. For example, thinking about how happy one feels (Bijttebier et al., 2012). Whereas dampening has been found to be more maladaptive (i.e., higher levels of dampening are related to more concurrent depressive symptoms), focusing is an adaptive emotion regulation strategy with low levels of focusing predicting more depressive symptoms both concurrently and longitudinally (Bijttebier et al., 2012).

### Insecure Attachment, Emotion Regulation, and Depressive Symptoms

Given the link between attachment and emotion regulation, and between emotion regulation and depressive symptoms, scholars proposed that emotion regulation strategies mediate the association between attachment and depressive symptoms (e.g., Brumariu & Kerns, 2010). Scarce cross-sectional research in adolescence provides support for this proposition (Malik et al., 2015). For example, some studies found evidence for rumination in general or brooding in specific mediating the association between attachment anxiety and depressive symptoms, and for suppression of negative emotions mediating the link between attachment avoidance and depressive symptoms (e.g., Brenning et al., 2012; Van de Walle et al., 2016). However, the cross-sectional nature of these studies did not allow to examine a temporal pathway and cross-sectionally tested mediation effects are at risk to provide biased estimates (e.g., Maxwell et al., 2011). Moreover, no previous studies tested whether positive affect regulation strategies mediate the association between insecure attachment and depressive symptoms.

## Current Study

Since prior research testing emotion regulation strategies as mediator in the association between attachment and depressive symptoms was cross-sectional and did not examine strategies for regulating positive affect, to date it remains unknown whether insecure attachment styles predict less adaptive negative and positive affect regulation strategies, which in turn enhance the risk to develop depressive symptoms in adolescence. To fill this knowledge gap, the current study longitudinally tested whether the association between attachment and change in depressive symptoms over time is explained by strategies for regulating negative affect (brooding and reflection) and positive affect (dampening and focusing) in adolescence. Based on the hypothesis that anxious attachment is linked to hyperactivation of negative emotions and minimization of positive emotions, the first tested prediction was that more anxiously attached adolescents develop more depressive symptoms via increased brooding on negative affect, increased dampening of positive affect and decreased focusing on positive affect. Based on the hypothesis that avoidant attachment is associated with deactivating emotion regulation strategies, the second tested prediction was that more avoidantly attached adolescents develop more depressive symptoms through increased dampening of positive affect and decreased focusing on positive affect.

## Methods

### Participants

The sample included 1706 children (53% girls) aged 9 to 17 years (*M* = 12.78, SD = 1.54) at Time 1 (T1). Seventy-seven percent of children had the Belgian nationality or originated from Belgium, 2% had the Dutch nationality, 3% had another nationality or country of origin, and for 18% data on nationality or country of origin was missing. Children participated at T1, Time 2 (T2, *M*_age_ = 13.74, SD_age_ = 1.55), and Time 3 (T3, *M*_age_ = 14.68, SD_age_ = 1.49) of two larger, four-wave longitudinal studies (Study 1: *n* = 1549, see also: Danneel et al., 2019; Nelis et al., 2016; Study 2: *n* = 157, see also: Van de Walle et al., 2016, 2017). Both studies used the same measures and similar set-ups.

### Measures

#### Depressive symptoms

The Children’s Depression Inventory (CDI; Kovacs, 2003; Dutch translation by Timbremont & Braet, 2002) is a questionnaire consisting of 27 items to assess cognitive, affective and behavioral depressive symptoms one has experienced in the past two weeks. Children completed the CDI at T1 and T3, and rated each item (e.g., *I feel like crying every day/many days/sometimes*) on a 3-point scale, going from 0 to 2. The scores were averaged across the 27 items to compose a mean score on the CDI. A higher score indicates a higher severity of self-reported depressive symptoms. The CDI proved to be a reliable and valid instrument in previous research (Kovacs, 2003). In the current study, the internal consistency of self-reported depressive symptoms using the CDI was very good at T1 (*α* = 0.86) and T3 (*α* = 0.87).

#### Attachment anxiety and avoidance

Children completed a shortened version of the Experiences in Close Relationships Scale- Revised (ECR-RC; Brenning et al., 2011, 2014) at T1 to measure attachment anxiety and attachment avoidance. The shortened version consists of 12 items, which operationalize attachment anxiety (6 items, e.g., *I am worried that my mother might want to leave me*) and attachment avoidance (6 items, e.g., *I prefer not to get too close to my mother*). Children scored each item on a 7-point Likert scale, going from 1 (*strongly disagree*) to 7 (*strongly agree*). Item scores were averaged to compose a mean for both attachment anxiety and attachment avoidance. Previous research showed good reliability and validity (both construct and predictive validity) for the long and the short ECR-RC (Brenning et al., 2011, 2014). The internal consistency in this study was good for attachment anxiety (*α* = 0.85) and attachment avoidance (*α* = 0.80).

#### Brooding and reflection

The Children’s Response Styles Questionnaire- Extended (CRSQ-ext.; Verstraeten et al., 2010) was administered at T2 to assess the extent to which children used brooding and reflection as coping strategies. Items were scored on a 4 point-Likert scale ranging from 1 (*almost never*) to 4 (*almost always*). The subscale brooding consists of 5 items (e.g., *When I am sad, I think about a recent situation wishing it had gone better*). Item scores were averaged to compute a mean score for brooding. The internal consistency for brooding was good (*α* = 0.80). The subscale reflection consists of 5 items (e.g., *When I am sad, I go away by myself and think about why I feel this way*). A mean score for reflection was calculated across 5 items. The internal consistency for reflection was good (*α* = 0.76).

#### Dampening and focusing

The Responses to Positive Affect Questionnaire for Children (RPA-C; Bijttebier et al., 2012) was administered at T2 to assess the child’s response styles to positive affect, more specifically dampening and focusing. The original RPA consists of 17 items which are scored on a four-point scale ranging from 1 (*almost never*) to 4 (*almost always*). Conform other studies that used the Dutch version of the RPA-C (e.g., Bijttebier et al., 2012; Verstraeten et al., 2012), one item in the dampening subscale was excluded from analyses due to its poor factor loading on the scale. Consequently, a total of 16 items were included, organized in three subscales: dampening (7 items, e.g., *My streak of luck is going to end soon*), self-focused positive rumination (4 items, e.g., *I am achieving everything*) and emotion-focused positive rumination (5 items, e.g., *Think about how happy you feel*). Consistent with previous research (Bijttebier et al., 2012), a parsimonious model with one factor of positive rumination was adopted in the current study and named focusing. The internal consistency was very good for dampening (*α* = 0.83) and focusing (*α* = 0.86).

### Procedure

Participant recruitment for both studies occurred via schools. For Study 1 (see e.g., Nelis et al., 2016), a total of 28 schools were randomly selected out of an exhaustive list with all school in Flanders, the northern part of Belgium. Eventually, seven schools agreed to participate. All pupils from grades 5 to 9 were sent home with a letter describing the aim and the procedure of the study. Parental permission was asked through passive consent. Adolescents provided active informed consent. Participants filled out paper-and-pencil questionnaires in their classroom during regular school hours. A research assistant was present during the test sessions to answer questions and to emphasize the voluntary and anonymous character of participation. Adolescents were informed that they could discontinue their participation at any time. After each assessment wave, cinema tickets were given to thank participating pupils. Data collection took place from February 2013 to May 2015.

For Study 2 (see e.g., Van de Walle et al., 2016), participant recruitment occurred through the distribution of flyers in grades 4 to 6 of schools in Flanders. Children and their mothers who were willing to participate in the study, were invited by phone or e-mail for an assessment at the research unit. Active informed consent was obtained from both mother and child upon arrival at the research unit. The assessment was part of a broader study focusing on attachment, cognitive vulnerabilities and depression. A research assistant was present during the test sessions. The currently used questionnaires were completed by children on paper. Each mother-child dyad was rewarded for participation after each measurement wave with cinema tickets and by including them in a lottery to win a larger price. Data collection took place from April 2013 to June 2015. For both studies, ethical approval was granted by the Social and Societal Ethics Committee of KU Leuven (ethical approval numbers: S55360 and S54874).

### Analyses

All analyses that were performed for the current study are reported below, i.e., no additional analyses were performed that included other variables or that were not reported because of lack of significance.

#### Preliminary analyses

Preliminary analyses were performed in SPSS version 26. Descriptives and bivariate Pearson correlations among the study’s main variables, and with T1 age were examined. T-tests were performed to assess sex differences on the main study variables. As not all variables conformed to a normal distribution, Spearman correlations and Mann–Whitney *U* tests were also conducted to see whether findings converged.

#### Main analyses

The lavaan package in R (Rosseel, 2012) was used to carry out two multiple mediation analyses with T1 attachment (anxiety or avoidance) as predictor variable, T3 depressive symptoms as criterion variable, and T2 emotion regulation strategies as mediating variables, with the models controlled for T1 depressive symptoms, T1 age, and sex. T2 emotion regulation strategies were allowed to covary with one another. The maximum likelihood (ML) estimator was used. Standard errors were bootstrapped (10,000 samples) because some variables did not adhere to a normal distribution. Effects for which *p* values < 0.05 were considered significant.

#### Sensitivity and exploratory analyses

To test the robustness of the results, the two multiple mediation analyses were repeated while additionally controlling for the other attachment dimension (i.e., in the model with attachment anxiety as predictor, the model was controlled for attachment avoidance and vice versa). Additionally, as sex differences in adolescent depression are frequently reported in the literature (e.g., Salk et al., 2017), differences between girls and boys were explored by running multi-group mediation analyses (controlling for T1 depressive symptoms and T1 age).

## Results

### Preliminary Analyses

Descriptive statistics and Pearson correlations among the main variables are shown in Table 1. Age at T1 was positively associated with T1 attachment avoidance, *r* = 0.20, *p* < 0.001, and with T1 depressive symptoms, *r* = 0.15, *p* < 0.001, but not with any of the other main study variables, *r*s between −0.04 and 0.06, *p*s > 0.07. There was no difference between sexes on attachment anxiety, *t*(1662) = 00.54, *p* = 0.59, but boys did show higher levels of attachment avoidance than girls, *t*(1674) = 2.50, *p* < 0.05. Moreover, girls showed higher levels of brooding, *t*(1305) = −6.22, *p* < 0.001, reflection, *t*(1322.22) = −5.96, *p* < 0.001, and dampening, *t*(1311.30) = −2.23, *p* < 0.05, and lower levels of focusing, *t*(1020) = 2.75, *p* < 0.01, compared to boys. Additionally, girls reported higher levels of depressive symptoms both at T1, *t*(1649.98) = −5.25, *p* < 0.001, and T3, *t*(1058) = −2.70, *p* < 0.01. Spearman correlation analyses and Mann–Whitney *U* tests revealed similar results in terms of significance and direction, except for the association between age at T1 and T3 depressive symptoms, which became significant, *r*_*s*_ = 0.07, *p* < 0.05, the association between attachment avoidance and reflection which became non-significant, *r*_*s*_ = 0.03, *p* = 0.29, and the sex effect for dampening which became non-significant, *U* = 204,499, *p* = 0.09.

**Table 1:** Descriptive statistics of and Pearson correlations among attachment, emotion regulation, and depressive symptoms based on full-cases data

	1.	2.	3.	4.	5.	6.	7.	8.
1. T1 attachment anxiety	1							
2. T1 attachment avoidance	0.42***	1						
3. T2 brooding	0.21***	0.15***	1					
4. T2 reflection	0.13***	0.06*	0.57***	1				
5. T2 dampening	0.22***	0.13***	0.48***	0.32***	1			
6. T2 focusing	−0.06*	−0.14***	0.02	0.13***	0.09**	1		
7. T1 depressive symptoms	0.41***	0.38***	0.37***	0.18***	0.32***	−0.21***	1	
8. T3 depressive symptoms	0.28***	0.25***	0.41***	0.25***	0.38***	−0.20***	0.52***	1
*M*	1.60	3.06	2.17	1.98	1.89	2.59	0.33	0.30
SD	0.94	1.37	0.76	0.65	0.66	0.62	0.24	0.23

Ns vary due to missing data

*p < 0.05; **p < 0.01; ***p < 0.001

Seventeen percent of all values on the main study variables were missing. Little’s MCAR test indicated that the data were not missing completely at random, *χ*^2^(185) = 283.55, *p* < 0.001. Missing at Random (MAR) cannot be tested statistically, and we assumed data to be MAR. Full information maximum likelihood (FIML) was used to handle missing data in further analyses.

### Indirect Associations Between Attachment Anxiety and Depressive Symptoms via Emotion Regulation

**Fig. 1:**
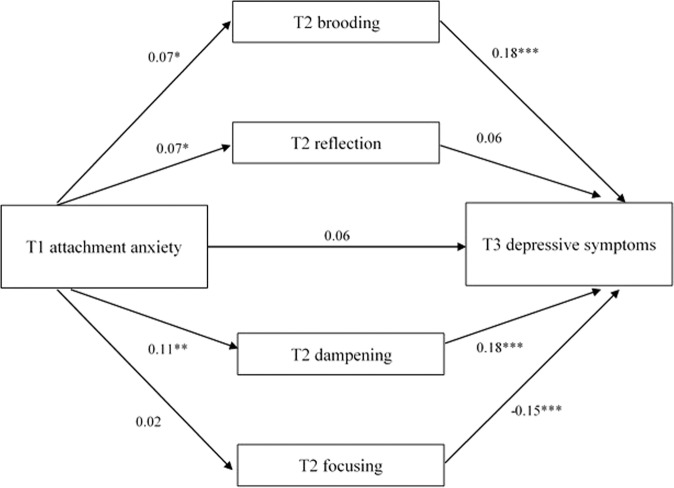
Multiple mediation model of the association between T1 attachment anxiety and T3 depressive symptoms via T2 emotion regulation strategies brooding, reflection, dampening, and focusing, with the model controlled for T1 depressive symptoms, T1 age and sex. Covariances between the T2 emotion regulation strategies were included in the model, but not depicted here. The standardized regression coefficients are mentioned along the paths. *p < 0.05, **p < 0.01, ***p < 0.001

The multiple mediation model with attachment anxiety as predictor is presented in Fig. 1. There was no direct effect of attachment anxiety at age 12 on depressive symptoms at age 14. Attachment anxiety at age 12 positively predicted brooding, reflection, and dampening at age 13. Moreover, higher levels of brooding and dampening, and lower levels of focusing at age 13 were associated with more depressive symptoms at age 14. There were significant indirect effects of attachment anxiety on depressive symptoms via brooding, *β* = 0.01, *p* < 0.05, and dampening, *β* = 0.02, *p* < 0.01, indicating that more anxiously attached children showed higher increases in depressive symptoms over time through enhanced levels of brooding and dampening. There were no indirect effects via reflection and focusing, *p*s > 0.18 (see Table 2).

**Table 2:** Unstandardized estimates and standard errors (SE, based on 10,000 bootstrap resamples) for the indirect effects of T1 attachment (anxiety and avoidance) on T3 depressive symptoms through T2 brooding, reflection, dampening, and focusing, with the model controlled for T1 depressive symptoms, T1 age and sex

	Unstandardized estimate	SE
T1 attachment anxiety		
T2 brooding	0.003*	0.002
T2 reflection	0.001	0.001
T2 dampening	0.005**	0.002
T2 focusing	−0.001	0.001
T1 attachment avoidance		
T2 brooding	0.001	0.001
T2 reflection	0.000	0.000
T2 dampening	0.001	0.001
T2 focusing	0.002*	0.001

*p < 0.05; **p < 0.01

### Indirect Associations Between Attachment Avoidance and Depressive Symptoms via Emotion Regulation

**Fig. 2:**
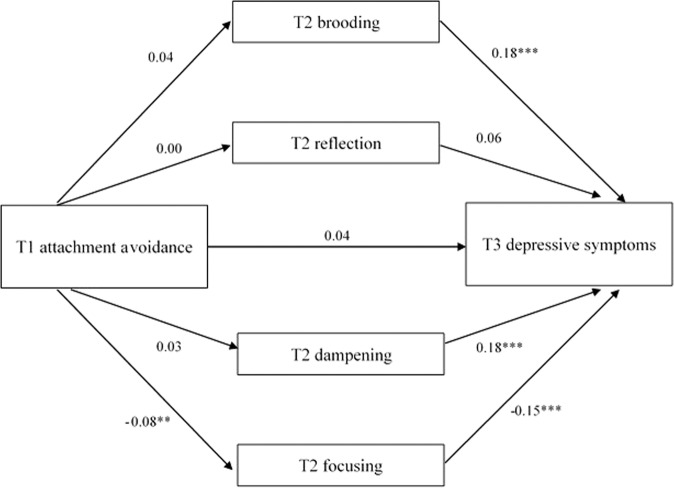
Multiple mediation model of the association between T1 attachment avoidance and T3 depressive symptoms via T2 brooding, reflection, dampening, and focusing, with the model controlled for T1 depressive symptoms, T1 age and sex. Covariances between the T2 emotion regulation strategies were included in the model, but not depicted here. The standardized regression coefficients are mentioned along the paths. *p < 0.05, **p < 0.01, ***p < 0.001

The multiple mediation model with attachment avoidance as predictor is presented in Fig. 2. There was no direct effect of attachment avoidance on depressive symptoms. Attachment avoidance at age 12 negatively predicted focusing at age 13. Again, brooding and dampening positively predicted depressive symptoms, and focusing negatively predicted depressive symptoms. The indirect effect via focusing was significant, *β* = 0.01, *p* < 0.05 (see Table 2). More avoidantly attached children developed more depressive symptoms over time via decreased levels of focusing.

### Sensitivity and Exploratory Analyses

The results for the multiple mediation model with attachment anxiety as predictor were maintained when attachment avoidance was added as a covariate in the model to control for the overlap between the two attachment dimensions, except for the indirect effect via brooding which became borderline significant, *β* = 0.01, *p* = 0.05. Moreover, the results for the mediation model with attachment avoidance as predictor were maintained when the model was controlled for attachment anxiety.

**Fig. 3:**
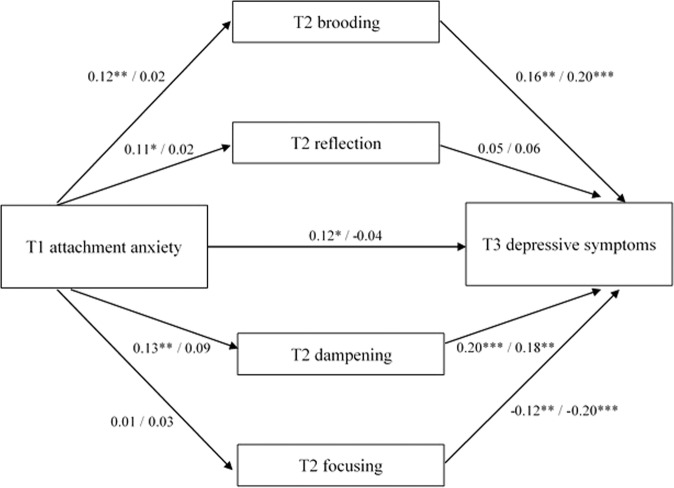
Multiple mediation models of the association between T1 attachment anxiety and T3 depressive symptoms via T2 emotion regulation strategies brooding, reflection, dampening, and focusing for boys and girls separately. Standardized coefficients for girls are presented left of the slashes, coefficients for boys are presented right of the slashes. The models were controlled for T1 depressive symptoms and T1 age. Covariances between the T2 emotion regulation strategies were included in the model, but not depicted here. *p < 0.05, **p < 0.01, ***p < 0.001

**Fig. 4:**
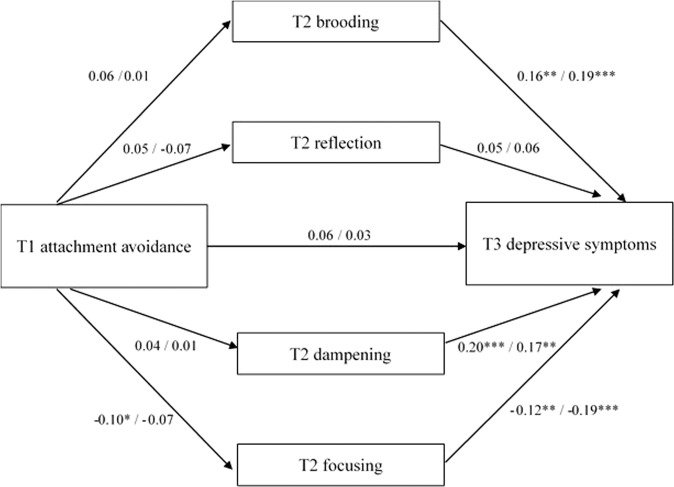
Multiple mediation models of the association between T1 attachment avoidance and T3 depressive symptoms via T2 emotion regulation strategies brooding, reflection, dampening, and focusing for boys and girls separately. Standardized coefficients for girls are presented left of the slashes, coefficients for boys are presented right of the slashes. The models were controlled for T1 depressive symptoms and T1 age. Covariances between the T2 emotion regulation strategies were included in the model, but not depicted here. *p < 0.05, **p < 0.01, ***p < 0.001

The models resulting from the exploratory multi-group mediation analysis distinguishing between girls and boys with attachment anxiety as predictor are presented in Fig. 3. The direct path from attachment anxiety to depressive symptoms significantly differed between groups, *β* = 0.17, *p* < 0.05. In girls, attachment anxiety positively predicted depressive symptoms, *β* = 0.12, *p* < 0.05, whereas for boys there was no significant association, *β* = −0.04, *p* = 0.38. Although the models showed that the direct paths from attachment anxiety to brooding, reflection and dampening, and the indirect effects via brooding and dampening only reached significance in the group of girls, comparison of the direct and indirect effects indicated no significant differences between girls and boys for any of the effects, *p*s > 0.12. The exploratory multi-group mediation analysis with attachment avoidance as predictor indicated no differences between sexes for any of the direct or indirect effects, *p*s > 0.06. See Fig. 4 for the models for girls and boys separately.

## Discussion

Past research testing emotion regulation strategies as mediator in the association between attachment and depressive symptoms in adolescence was cross-sectional and did not examine strategies for regulating positive affect. Lack of such research raised the risk that this emotion-regulation hypothesis of attachment theory got prematurely accepted and that the understanding of the emotion regulation strategies relevant for the link between attachment and depressive symptoms was too narrow. The current study aimed to fill these existing research gaps and examined whether the association between attachment and development of depressive symptoms over time is mediated by strategies for regulating negative and positive affect. The results from multiple mediation analyses showed that attachment anxiety and attachment avoidance were associated with depressive symptoms via emotion regulation strategies. For the association between attachment anxiety and depressive symptoms, strategies for the regulation of both negative and positive affect were important. For the association between attachment avoidance and depressive symptoms, less adaptive regulation of positive affect emerged as a unique underlying mechanism.

### Attachment Anxiety-Related Emotion Regulation and the Development of Depressive Symptoms

More anxiously attached children reported more brooding, which mediated the association between attachment anxiety and depressive symptoms. This finding fits well with theory and prior research. Theoretically, brooding can serve to elicit attention and care from an inconsistently sensitive and responsive caregiver and is therefore an emotion regulation strategy that fits with more anxiously attached children’s relational goals even though it increases their risk to develop depressive symptoms (Mikulincer et al., 2003). Past cross-sectional research already found that brooding mediated the relation between attachment anxiety and depressive symptoms concurrently (Van de Walle et al., 2016). The current study is the first to provide evidence that brooding is relevant to understand the longitudinal association between attachment anxiety and development of depressive symptoms in adolescence. Of note, when controlling for attachment avoidance, the indirect effect of brooding failed to reach significance. This suggests that this indirect effect may not be specific to attachment anxiety, but may be driven by general insecure attachment.

Additionally, more anxiously attached children reported more dampening of positive affect which mediated the association between attachment anxiety and depressive symptoms. This effect remained significant after controlling for attachment avoidance. More anxiously attached children have been proposed to be motivated to heighten their negative affect in the service of eliciting parental proximity and care (Cassidy, 1994). Dampening of positive affect might further serve this attachment-related goal. Accordingly, previous studies in adults already reported more dampening of positive affect for more anxiously attached individuals (Gentzler et al., 2010, 2015). However, no previous research assessed in adolescents whether dampening of positive affect relates to attachment anxiety, or whether dampening mediates the relation between attachment anxiety and the development of depressive symptoms. The current study thus adds to the existing literature by indicating that more anxiously attached adolescents are more at risk for developing depressive symptoms, not only because they heighten their negative affect (brooding), but additionally because they minimize positive affect (dampening).

Furthermore, results showed that reflection and focusing did not mediate the association between attachment anxiety and depressive symptoms. As for reflection, this can be attributed to the fact that reflection did not relate to changes in depressive symptoms. This observation fits with previous research reporting mixed findings on the association between reflection and depressive symptoms in terms of direction and significance (Treynor et al., 2003; Verstraeten et al., 2010). Concerning focusing, the direct association between attachment anxiety and focusing was not significant. Previous studies in adults found that more anxiously attached individuals were less inclined to maximize positive affect, or found no attachment anxiety-related differences in up-regulating positive affect (Gentzler et al., 2010, 2015). One explanation for the currently found insignificant association might be that more anxiously attached children have contrasting motivational goals due to which some of them focus less on positive affect whereas others focus more on positive affect. On the one hand, more anxiously attached children might feel motivated to focus less on positive affect in the service of heightening negative affect and eliciting care. On the other hand, they might feel motivated to maximize positive emotions as an alternative strategy to enhance closeness to the attachment figure (Mikulincer & Shaver, 2007). Such contrasting motivations could have suppressed the association between anxious attachment and focusing on positive affect.

Finally, exploratory multi-group analyses showed no significant sex-related differences for any of the indirect effects, indicating that the underlying mechanisms of the association between attachment anxiety and depressive symptoms may be similar for girls and boys. Taken together, the current results suggest that the association between attachment anxiety and depressive symptoms is mediated by the use of maladaptive emotion regulation strategies (brooding and dampening), rather than the non-use of adaptive emotion regulation strategies (focusing).

### Attachment Avoidance-Related Emotion Regulation and the Development of Depressive Symptoms

More avoidantly attached adolescents reported to focus their attention less on the experience of positive affect, which in turn related to greater relative increases in depressive symptoms. This effect remained significant after controlling for attachment anxiety. This finding conforms with the prediction that attachment avoidance is related to the inability to benefit from positive affect (Mikulincer et al., 2003). The inability to use positive emotions as a resource is a unique mechanism that puts children at risk to develop depressive symptoms, over and above the use of strategies for regulating negative emotions (Bijttebier et al., 2012). While most prior mechanism research focused specifically on maladaptive factors underlying the association between avoidant attachment and depressive symptoms, the current study suggests that adolescents’ non-use of the adaptive strategy to focus on positive affect is important to understand the link between attachment avoidance and the development of depressive symptoms in adolescence.

The current study found no evidence that the assessed negative affect regulation strategies (i.e., brooding and reflection), or dampening of positive affect underlie the link between attachment avoidance and depressive symptoms in adolescence. These three affect regulation strategies were unrelated to attachment avoidance in the multiple mediation model, and reflection was also not related to depressive symptoms. The finding that attachment avoidance was not associated with dampening of positive affect may be considered somewhat unexpected, because theory suggests that more avoidantly attached children learn in their attachment relationships to suppress emotions in order to maximize interpersonal distance and to avoid further decay in insecure relationships (Cassidy, 1994). Moreover, research in adults indicated a positive association between avoidant attachment and dampening (Gentzler et al., 2010). However, the latter finding was observed in a substantially smaller number of adults (*N* = 119), while the size of the current sample might allow to draw more reliable conclusions. Moreover, it is not unlikely that current findings diverge as the current study focused on a younger sample and measured positive affect regulation in a different way. Results from exploratory analyses indicated no sex-related differences for any of the indirect effects in the association between attachment avoidance and depressive symptoms, suggesting that the underlying mechanisms are similar for girls and boys.

Finally, the current observation that maladaptive emotion regulation strategies do not explain avoidantly attached adolescents’ risk to develop depressive symptoms fits with prior research. Many studies struggle to find strong and convincing evidence that the link between attachment avoidance and the development of psychopathology is mediated by risk-related mechanisms (see e.g., Malik et al., 2015). This makes sense as avoidant attachment is generally considered a more adaptive attachment style that is overall less linked to psychopathology than anxious attachment (Mikulincer & Shaver, 2016). The current findings might indicate that the real mental health risk for more avoidantly attached adolescents is that they can benefit less from positive experiences in their life.

### Limitations

The present study’s findings should be considered within the context of its limitations. First, a community sample was tested and overall children reported to be relatively securely attached and many children did not show clinical levels of depression (i.e., 89% at T3). Although this is in line with the expected prevalence at population level (Timbremont & Braet, 2005), this limits the generalizability of the currently reported effects to adolescents with more severe depressive symptoms or higher levels of insecure attachment. Therefore, future research including a sample with a wider range of depressive symptoms, attachment anxiety, and attachment avoidance would be valuable to assess whether the current results can be replicated. Nevertheless, research in adolescents falling below the clinical range of depression remains important to gain insight into the full range of developmental risk and resilience factors related to depression (Cicchetti & Rogosch, 2002). Second, there was limited demographic information about the current sample and reporting on, for instance, ethnicity diversity was not possible. Therefore, it remains for future studies to explore whether the current findings generalize across ethnicities. Third, all of the used measures were self-reported, which leads to the risk of shared method variance contributing to the findings. Therefore, a multi-method approach would be beneficial for future research. Moreover, future studies may consider using the Inventory of Parent and Peer Attachment, as this measure has been evaluated as having the most potential to measure attachment in adolescence (Jewell et al., 2019). However, the currently used ECR-RC allows distinguishing between anxious and avoidant attachment styles, which was crucial to evaluate the premise that different insecure attachment styles relate to depressive symptoms via different types of emotion regulation strategies (i.e., more hyper- versus deactivating strategies). Finally, the current study only focused on the mother-child relationship, whereas other attachment relationships, e.g., the father-child relationship, might very well be important for emotion regulation and the development of depressive symptoms (Agerup et al., 2015). Therefore, future studies should aim to include other or multiple attachment relationships. Nevertheless, research does suggest that for the majority of children this age, the mother serves as the primary attachment figure (Grossmann et al., 2008; Kerns et al., 2006).

### Implications

The current study offers implications for developmental research and for clinical practice. Longitudinal associations between attachment, emotion regulation and depressive symptoms were found. These findings contribute to the understanding of the development of depressive symptoms in adolescence by indicating that insecure attachment is a risk factor for the development of more maladaptive or less adaptive emotion regulation strategies, which subsequently serve as a risk factor for developing depressive symptoms. However, it cannot be concluded whether these associations are causal. Therefore, future research should aim to examine whether enhancing or restoring secure attachment can prevent individuals from developing maladaptive emotion regulation strategies and prevent depressive symptoms later on. Moreover, the current finding that attachment avoidance is related to the development of depressive symptoms via decreased use of the adaptive emotion regulation strategy focusing calls for more research into resilience-related factors underlying the association between attachment avoidance and depression. Further suggesting that the link between avoidant attachment and depressive symptoms results from a lack of resilience-related resources, a recent study found that more avoidantly attached adolescents experienced less trait gratitude (i.e., the positive feeling of appreciation for what one receives), due to which they were more vulnerable to develop depressive symptoms (Scott et al., 2020). Research into resilience-related factors may thus provide more insight into the developmental processes contributing to depression for more avoidantly attached individuals. Finally, the current findings suggest that future research should include strategies for regulation of both negative and positive affect as both may contribute to the ontogeny of depression over time in more insecurely attached adolescents. Future research may also want to consider other emotion regulation processes that may underlie to the association between attachment and depressive symptoms, such as distraction or acceptance (Cracco et al., 2015).

Clinically, there is an increasing awareness that it is important to focus on emotion regulation strategies in the screening, prevention and treatment of depression (e.g., Berking & Wupperman, 2012). However, the current results suggest that it might additionally be relevant to account for the attachment background of children. Prevention efforts may need to address emotion regulation within the parent-child attachment relationship. Concerning treatment, it has been suggested that attachment relationships can also function as a priming context that reactivate old emotion regulation strategies (Bosmans, 2016; Bosmans, Bakermans-Kranenburg et al., 2020). Hence, it is not unlikely that existing emotion regulation treatments are less effective for less securely attached adolescents. It might be that these adolescents are less able to apply their newly developed emotion regulation skills at home where their emotion regulation behavior is directly affected by fear for rejection or frustration about the unavailability of parents to scaffold their attempts to regulate distress (Kobak & Bosmans, 2019). Future research should evaluate whether strengthening the parent-child attachment relationship could be a valuable approach for children with maladaptive emotion regulation strategies. To date, there is a dearth of interventions focusing on repairing secure attachment in parent-adolescent relationships (for a notable exception see Diamond et al., 2014). There is a need to develop such interventions further to test whether clinicians should primarily target children’s emotion regulation strategies, the underlying attachment relationships, or both, to protect children from further developing depressive symptoms.

## Conclusion

Attachment theory hypothesizes that insecurely attached adolescents are at increased risk to develop depressive symptoms via maladaptive emotion regulation strategies. Although longitudinal tests are critical to support this premise, they were missing to date. Moreover, attachment theory and research mainly focused on negative affect regulation and ignored strategies for the regulation of positive affect. Therefore, the current study tested whether insecure attachment is associated with the longitudinal development of depressive symptoms through the regulation of negative and positive affect. The results showed that more anxiously attached adolescents developed more depressive symptoms over time via increased brooding on negative affect and dampening of positive affect. Additionally, more avoidantly attached adolescents developed more depressive symptoms via decreased focusing on positive affect. The current study thus contributes to the understanding of depression development in adolescence by providing first longitudinal empirical support for attachment theory’s hypothesis that emotion regulation explains the development of depressive symptoms in more insecurely attached adolescents. Importantly, strategies for regulating negative affect as well as strategies for regulating positive affect can uniquely put more insecurely attached adolescents at risk for the development of depressive symptoms. It is recommended that future attachment research not only considers risk-related mediators of the attachment-psychopathology link, but also consider resilience-related mediators. For attachment avoidance specifically, research into resilience-related mediators may prove highly relevant as most studies, including the current study, show that it is hard to find mediating effects of risk-related factors. Finally, clinical practice may need to address emotion regulation strategies as well as the parent-child attachment relationship to enhance the effectiveness of depression interventions.
